# The Utility of Sentinel Lymph Node Biopsy in Papillary Thyroid Carcinoma with Occult Lymph Nodes

**DOI:** 10.1371/journal.pone.0129304

**Published:** 2015-06-05

**Authors:** Xingqiang Yan, Ruichao Zeng, Zhaosheng Ma, Chengze Chen, Endong Chen, Xiaohua Zhang, Feilin Cao

**Affiliations:** 1 Department of Surgical Oncology, Taizhou Hospital, Wenzhou Medical University, Linhai, Zhejiang Province, People’s Republic of China; 2 Department of Surgical Oncology, The First Affiliated Hospital, Wenzhou Medical University, Wenzhou, Zhejiang Province, People’s Republic of China; Uppsala University, SWEDEN

## Abstract

**Background:**

The sentinel lymph node (SLN) is defined as the first draining node from the primary lesion, and it has proven to be a good indicator of the metastatic status of regional lymph nodes in solid tumors. The aim of this study was to evaluate the clinical application of SLN biopsy (SLNB) in papillary thyroid carcinoma (PTC) with occult lymph nodes.

**Methods:**

From April 2006 to October 2012, 212 consecutive PTC patients were treated with SLNB using carbon nanoparticle suspension (CNS). Then, the stained nodes defined as SLN were collected, and prophylactic central compartment neck dissection (CCND) followed by total thyroidectomy or subtotal thyroidectomy were performed. All the samples were sent for pathological examination.

**Results:**

There were 78 (36.8%) SLN metastasis (SLNM)-positive cases and 134 (63.2%) SLNM-negative cases. The sensitivity, specificity, positive and negative predictive values, and false-positive and false-negative rates of SLNB were 78.8%, 100%, 100%, 84.3%, 0%, and 21.2%, respectively. The PTC patients with SLNM were more likely to be male (48.2% vs. 32.7%, p = 0.039) and exhibited multifocality (52.6% vs. 33.3%, p = 0.025) and extrathyroidal extension (56.7% vs. 33.5%, p = 0.015). A greater incidence of non-SLN metastases in the central compartment was found in patients with SLNM (41/78, 52.6%) than in those without SLNM (21/134, 15.7%; p < 0.05). However, the SLNM-negative PTC patients with non-SLN metastases were more likely to be male (37.9% vs. 9.5%, p < 0.05).

**Conclusions:**

The application of SLNB using CNS is technically feasible, safe, and useful, especially for male patients with co-existing multifocality and extrathyroidal extension. However, the sensitivity of SLNB must be improved and its false-negative rate reduced before it can be a routine procedure and replace prophylactic CCND. More attention should be paid to PTC patients (especially males) without SLNM for signs of non-SLN metastases.

## Introduction

Sentinel lymph nodes (SLNs), the first station in the lymphatic drainage basin, receive lymph flow from primary tumors and reflect the status of the remaining lymph nodes. In surgery, SLN biopsy (SLNB) has become a common method for treating several types of human malignant tumors, especially melanoma and breast cancer[1,2]. However, SLNB application to the treatment of papillary thyroid carcinoma (PTC) patients has not been thoroughly investigated.

PTC, including papillary microcarcinoma (PTMC, tumor size ≤ 1 cm), is the most common type of differentiated thyroid cancer and spreads predominantly via the lymphatics to the local draining lymph nodes. The metastases of occult lymph nodes are detected in approximately 27–90% of PTC cases after surgery and histologic examination[3–5]. Although PTC has a high rate of regional lymph node metastasis (LNM), the impact of regional LNM on the prognosis is unclear. Recent studies have indicated that regional LNMs increase the risk of locoregional recurrence and have an adverse effect on survival, especially in older patients (age>45 years)[4,6–8]. A study of 9,904 patients with PTC or follicular thyroid carcinoma indicated that LNM is a significant independent factor of poor prognosis based on multivariate analysis[9]. By contrast, other studies have demonstrated that occult LNM increases locoregional recurrence but does not affect disease-specific survival[10–12]. An investigation revealed that palpable lymph nodes in PTC patients should undergo therapeutic node dissection, whereas patients with occult lymph nodes do not benefit from prophylactic node dissection[13]. Therefore, appropriate management of PTC with overt or occult lymph nodes is very important for improving survival, decreasing regional recurrence and avoiding over-treatment.

In recent years, with the development of nanotechnology, nanocarbons have been widely used as a lymph node tracer in malignant tumors. An injection of carbon nanoparticle suspension (CNS) consists of nanosized carbon particles with an average diameter of 150 nm. Upon injection into the tissues around the tumor, nanocarbon particles are rapidly engulfed by macrophages. The particles then enter the lymphatic vessels and accumulate in the lymph nodes, staining them black. This technique has facilitated the vital staining of tumor-draining lymph nodes, and has been applied in the detection of SLNs in breast and gastric cancers[14,15].

PTC patients with demonstrated cervical LNM in the central or lateral compartment based on imaging studies or fine-needle aspiration cytology diagnosis should undergo therapeutic neck dissection; it is not recommended that patients with occult lymph nodes in the lateral compartment undergo prophylactic neck dissection [16]. However, the prophylactic central compartment (level VI) neck dissection (CCND) treatment for PTC patients with occult lymph nodes in the central compartment is still controversial because of its potential morbidity and indistinct benefits. Thus, we hypothesized that SLNB using CNS is a useful method that can replace prophylactic CCND to increase the benefit for PTC patients.

## Patients and Methods

### Ethics Statement

This study was approved by the Ethics Committee of The First Affiliated Hospital of Wenzhou Medical University, and written informed consent was obtained from all study subjects prior to enrollment. All data analyzed were anonymized.

### Patients

This prospective study was conducted on 212 patients with incipient thyroid cancer and a diagnosis of PTC. All patients underwent surgical treatment by SLNB and prophylactic CCND at our hospital from 1 April 2006 to 31 October 2012. They were diagnosed with PTC based on preoperative fine-needle aspiration cytology and postoperative pathology. We excluded patients who had preoperative evidence of cervical LNM based on physical examination or fine-needle aspiration cytology, ultrasound, and computed tomography. Patients with previous neck surgery or preoperative detectable lymph nodes were also excluded from our study.

### Surgical Procedure

**Fig 1:**
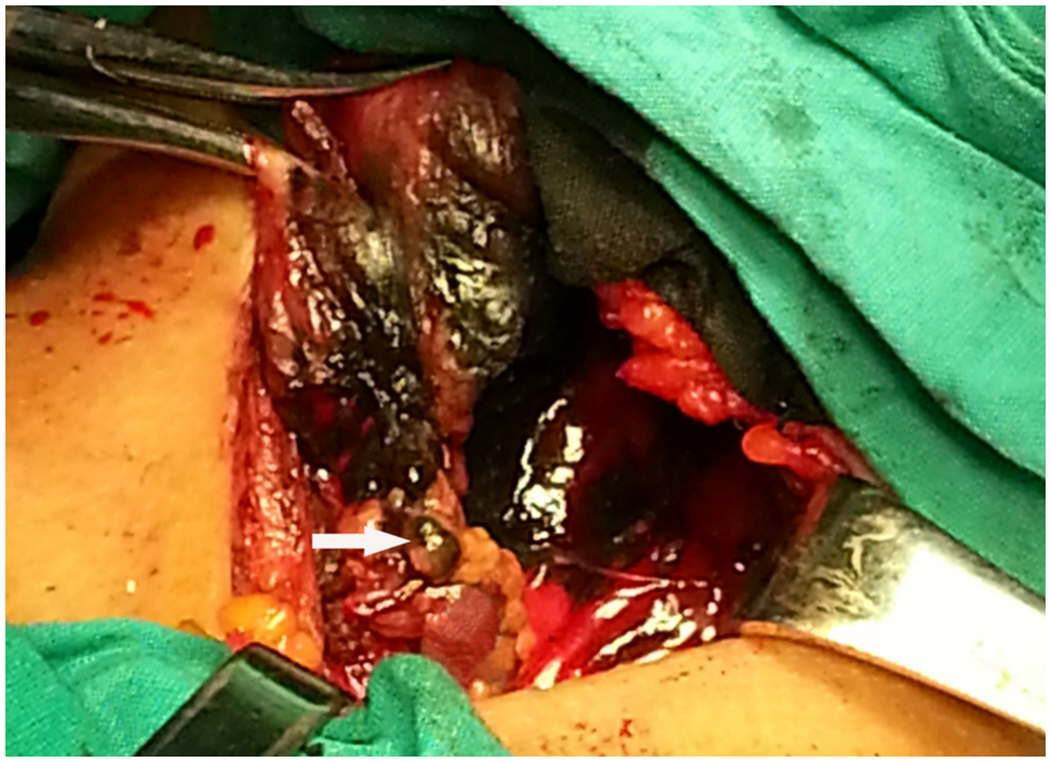
SLNB using CNS. The white arrow indicates the black-stained SLN in the central compartment. SLNB = sentinel lymph node biopsy; SLN = sentinel lymph node; CNS = carbon nanoparticle suspension.

All the surgery operations were performed by three experienced surgeons, who had performed more than one hundred thyroid surgeries per year within the past decade and had experience with thyroid SLN procedures. A transverse low-collar skin incision was followed by separation of the skin flap and a longitudinal incision in the linea alba cervicalis. Then, the thyroid capsule was carefully opened to completely expose the thyroid gland without injury to the capsule. The thyroid gland and the ipsilateral jugular vein were exposed. SLNB was performed according to the method described by Hao et al.[17]. Then, approximately 1 ml of a CNS (Chongqing Lummy Pharmaceutical Co., Ltd.) was injected using a 27-gauge needle into the parenchyma surrounding the primary tumor. For bilateral tumors or multiple tumors, the CNS was injected into those tumors that were suspected to be malignant. Within minutes, the stained lymphatic vessels from the primary tumor became apparent, and black-stained lymph nodes were identified via tracing the stained lymphatic vessels (Fig 1). We focused on black-stained lymph nodes in the central compartment. These black-stained lymph nodes, defined as SLNs, were carefully collected and sent to the pathology department for frozen and routine pathology. Subsequently, prophylactic CCND followed by total thyroidectomy or subtotal thyroidectomy were performed. The remaining non-stained lymph nodes in the central compartment, defined as non-SLNs, were sent for routine pathology. Each SLN was cut in half: one half was used for frozen pathology and the other half for routine pathology. All the samples were cut into 5-um-thick slices and stained by hematoxylin-eosin. LNM was defined as tumor tissue found in the slice. Several tumor markers were tested by an immunohistochemistry assay to improve the diagnosis. Criteria to identify malignant keratin-positive cells included strong cytokeratin immunoreactivity, anatomic location in nodal sinuses, and cytologic atypia similar to the primary carcinoma. All the diagnoses were made by two experienced pathologists, and all the final diagnoses were based on the routine pathology findings.

### Data Analysis

Descriptive statistics were used to analyze the characteristics of the patients and tumors. Univariate analysis and multivariate logistic regression analysis of the associations between the metastases of occult lymph nodes and several clinicopathological factors of the primary tumor were performed. The t-test and chi-square test were used for the measurements and count data, respectively. SPSS version (SPSS Inc., Chicago, IL, USA), version 19, was used in all of the statistical analyses, and statistical significance was defined as a p-value less than 0.05.

## Results

The characteristics of all patients are shown in Table 1 (Details are shown in S1 Table). In total, 212 patients underwent SLNB and prophylactic CCND; the mean age was 45.6 years (ranging from 21 to 72 years). The majority of the patients were female (156/212, 73.6%) and diagnosed with PTMC (143/212, 67.5%). Most PTCs do not present multifocality (38/212, 17.9%) and extrathyroidal extension (30/212, 14.2%). Nearly half of the patients were diagnosed with tumor encapsulation (88/212, 41.5%). A minority of patients presented high levels of serum TMAb and TGAb (51/212, 24.1%) and TSH (5/212, 2.4%) and developed thyroiditis (31/212, 14.6%). In the final pathologic reports, SLN metastases (SLNMs) were found in 78 (36.8%) patients and non-SLNs were found in 62 (29.2%) patients; 551 lymph nodes were detected in the SLNBs and 1621 lymph nodes were removed from the central compartment.

**Table 1:** Patient demographics and tumor characteristics (n = 212).

Characteristic	Value
Sex	
Male	56 (26.4%)
Female	156 (73.6%)
Age (years old)	
Mean + SD (range)	45.6±10.4(21–72)
>45	102 (48.1%)
≤45	110 (51.9%)
Tumor size (cm)	
>1	69 (32.5%)
≤1	143 (67.5%)
Multifocality	
Multifocal	38 (17.9%)
Unifocal	174 (82.1%)
TMAb, TGAb	
Abnormal	51 (24.1%)
Normal	161 (75.9%)
TSH	
Abnormal	5 (2.4%)
Normal	207 (97.6%)
Encapsulation	
Present	88 (41.5%)
Absent	124 (58.5%)
Extrathyroidal extension	
Present	30 (14.2%)
Absent	182 (85.8%)
Thyroiditis	
Present	31 (14.6%)
Absent	181 (85.4%)
SLN	
Positive	78 (36.8%)
Negative	134 (63.2%)
Non-SLN	
Positive	62 (29.2%)
Negative	150 (70.8%)
Number of nodes	
SLN (mean + SD)	551 (2.6±1.8)
Total-LN (mean + SD)	1621 (7.7±3.2)

TMAb = thyroid microsomal antibody; TGAb = thyroglobulin antibody; TSH = thyroid stimulating hormone; SLN = sentinel lymph node; Non-SLN = non-sentinel lymph node; Total-LN = total lymph nodes

**Fig 2:**
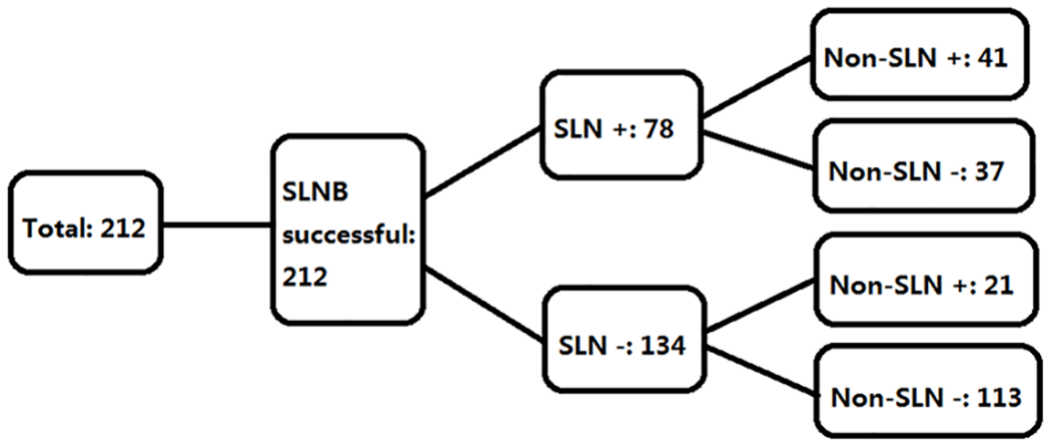
Overview of SLNB. SLNB = sentinel lymph node biopsy; SLN = sentinel lymph node; Non-SLN = non-sentinel lymph node.

The SLNB results are illustrated in Fig 2. In the present study, 78 patients were diagnosed with SLNM, and in 41 patients, further metastases in the non-SLN samples were detected. The remaining 134 patients were not diagnosed with SLNM, whereas 21 patients exhibited further metastases in the non-SLN samples. The SLNB results are shown in Table 2 Part A. The sensitivity, specificity, positive and negative predictive values, and false-positive and false-negative rates of SLNB were 78.8%, 100%, 100%, 84.3%, 0%, and 21.2%, respectively (Table 2 Part B). Among these patients, males had a significantly higher rate of SLNM compared with females (48.2% vs. 32.7%, p = 0.039), and multifocal PTC had a higher probability of SLNM compared with unifocal PTC (52.6% vs. 33.3%, p = 0.025). More SLNMs were found in PTC with extrathyroidal extension than in PTC without extrathyroidal extension (56.7% vs. 33.5%, p = 0.015). However, other factors such as patient age; tumor size; serum TMAb, TGAb and TSH levels; encapsulation; and thyroiditis did not show statistical significance for SLNM (Table 3). A multivariate analysis was performed to determine whether these factors were independently correlated with SLNM. Male gender (OR = 1.939, p = 0.043), multifocality (OR = 2.204, p = 0.035) and extrathyroidal extension (OR = 2.624, p = 0.033) were independently predictive of SLNM (Table 4). In addition, patients with SLNM (41/78) had a greater incidence of non-SLN compared to those without SLNM (21/134) (52.6% vs. 15.7%, p = 0.000) (Table 3).

**Table 2:** Results of SLNB using CNS in the central compartment.

A			
	SLN +	SLN -	Total
Central lymph node +	78	21	99
Central lymph node -	0	113	113
Total	78	134	212
B			
Characteristic		Value	
Detection rate		212/212 (100%)	
Sensitivity		78/99 (78.8%)	
Specificity		113/113 (100%)	
Positive predictive value		78/78 (100%)	
Negative predictive value		113/134 (84.3%)	
False-positive rate		0/113 (0%)	
False-negative rate		21/99 (21.2%)	
Accuracy		191/212 (90.1%)	

SLNB = sentinel lymph node biopsy; SLN = sentinel lymph node; CNS = carbon nanoparticle suspension

**Table 3:** Comparison of patients with and without SLN metastasis (n = 212).

	Metastases (n = 78)	Non-metastases (n = 134)	p
Sex			0.039*
Male	27 (48.2%)	29 (51.8%)	
Female	51 (32.7%)	105 (67.3%)	
Age (years old)			0.197
>45	33 (32.4%)	69 (67.6%)	
≤45	45 (40.9%)	65 (59.1%)	
Tumor size (cm)			0.272
>1	29 (42.0%)	40 (58.0%)	
≤1	49 (34.3%)	94 (65.7%)	
Multifocality			0.025*
Multifocal	20 (52.6%)	18 (47.4%)	
Unifocal	58 (33.3%)	116 (66.7%)	
TMAb, TGAb			0.937
Abnormal	19 (37.3%)	32 (62.7%)	
Normal	59 (36.6%)	102 (63.4%)	
TSH			0.160
Abnormal	0 (0.0%)	5 (100.0%)	
Normal	78 (37.7%)	129 (62.3%)	
Encapsulation			0.492
Present	30 (34.1%)	58 (65.9%)	
Absent	48 (38.7%)	76 (61.3%)	
Extrathyroidal extension			0.015*
Present	17 (56.7%)	13 (43.3%)	
Absent	61 (33.5%)	121 (66.5%)	
Thyroiditis			0.170
Present	8 (25.8%)	23 (74.2%)	
Absent	70 (38.7%)	111 (61.3%)	
No. of SLNs	217 (39.4%)	334 (60.6%)	
Mean + SD (range)	2.8±1.9	2.5±1.7	0.258
Non-SLN			0.000*
Metastasis	41 (66.1%)	21 (33.9%)	
Non-metastasis	37 (24.7%)	113 (75.3%)	

TMAb = thyroid microsomal antibody; TGAb = thyroglobulin antibody; TSH = thyroid stimulating hormone; SLN = sentinel lymph node; Non-SLN = non-sentinel lymph node;

* p<0.05

**Table 4:** Multivariate analysis of the clinicopathological factors for patients with SLN metastasis.

Factors	Odds Ratio	95% Confidence interval	p-value
Male sex	1.939	1.022–3.681	0.043*
Multifocality	2.204	1.058–4.589	0.035*
Extrathyroidal extension	2.624	1.082–6.366	0.033*

SLN = sentinel lymph node;

* p<0.05

Among the 134 patients who were not diagnosed with SLNM, 21 exhibited further metastases in the non-SLN samples. Male patients also had a significantly higher rate of non-SLN metastases compared with female patients (37.9% vs. 9.5%, p = 0.001). Age; tumor size; multifocality; serum TMAb, TGAb and TSH levels; encapsulation; extrathyroidal extension; thyroiditis; and mean number of SLNs were not significantly related to the non-SLNs (Table 5).

**Table 5:** Comparison of SLN-negative patients with and without non-SLN metastasis (n = 134).

	Metastases (n = 21)	Non-metastases (n = 113)	p
Sex			0.001*
Male	11 (37.9%)	18 (62.1%)	
Female	10 (9.5%)	95 (90.5%)	
Age (years old)			0.389
>45	9 (13.0%)	60 (87.0%)	
≤45	12 (18.5%)	53 (81.5%)	
Tumor size (cm)			0.156
>1	9 (22.5%)	31 (77.5%)	
≤1	12 (12.8%)	82 (87.2%)	
Multifocality			0.160
Multifocal	5 (27.8%)	13 (72.2%)	
Unifocal	16 (13.8%)	100 (86.2%)	
TMAb, TGAb			0.093
Abnormal	2 (6.3%)	30 (93.7%)	
Normal	19 (18.6%)	83 (81.4%)	
TSH			1.000
Abnormal	0 (0.0%)	5 (100.0%)	
Normal	21 (16.3%)	108 (83.7%)	
Encapsulation			0.163
Present	12 (20.7%)	46 (79.3%)	
Absent	9 (11.8%)	67 (88.2%)	
Extrathyroidal extension			1.000
Present	2 (15.4%)	11 (84.6%)	
Absent	19 (15.7%)	102 (84.3%)	
Thyroiditis			0.528
Present	2 (8.7%)	21 (91.3%)	
Absent	19 (17.1%)	92 (82.9%)	
No. of SLNs	47 (14.1%)	287 (85.9%)	
Mean + SD (range)	2.2±1.5	2.5±1.8	0.465

TMAb = thyroid microsomal antibody; TGAb = thyroglobulin antibody; TSH = thyroid stimulating hormone; SLN = sentinel lymph node; Non-SLN = non-sentinel lymph node;

* p<0.05

There were two types of postoperative complications in this study, including 2 hematomas and 20 cases of transient hypoparathyroidism. No cases of permanent recurrent nerve damaged and no complications or side effects related to the CNS were detected.

## Discussion

The role of routine CCND in the treatment of PTC remains unclear. It is generally agreed that therapeutic neck dissection should be performed to remove macroscopic LNM because this treatment reduces the chance of PTC persistence and recurrence[18]. However, there have been no randomized controlled trails to support the concept that routine prophylactic CCND affects the recurrence or survival rates of PTC patients with occult lymph nodes. In addition, prophylactic CCND may increase the risk of complications such as hypocalcemia and recurrent laryngeal nerve palsy. However, an additional operation may be necessary after recurrence, which will increase the risk of operative complications and medical costs. Therefore, the accurate identification of occult LNMs in PTC patients is helpful in the selection of an appropriate therapeutic strategy. An SLN is defined as the first draining node from the primary lesion and can indicate the metastatic status of regional lymph nodes. SLNB has proven to be a valuable surgical adjunct and has become the standard for the surgical approach to melanoma and breast cancer, helping to avoid unnecessary regional lymph node dissection[19]. Here, SLNB was introduced to assess the status of occult lymph nodes for PTC patients, and its utility was evaluated.

The radioisotope method and the dyeing method are usually used in SLNB. The radioisotope method has certain advantages, such as higher detection rates and no false-positive staining of the parathyroid gland[11]. However, the radioisotope method is complicated, time-consuming, and expensive and involves radiocontamination. In addition, there is no obvious difference in the sensitivity and specificity compared with that of the dyeing method [20,21]. In recent years, with the development of nanotechnology, nanocarbons have been widely used as a lymph node tracer in malignant tumors [14,15]. In this study, CNS was used as the dying method in SLNB. The detection rate of SLN was 100% (212/212), similar to the radioisotope method (96–100%) and higher than the methylene blue dye technique (79–91%)[20–23]. This technique was likely more efficient for detection because carbon absorption leads to more prolonged staining than with the traditional dye method using methylene blue. There were no differences in sensitivity or specificity, compared previous studies. Just as we showed in a previous study[17], this dye technique is helpful for thorough CCND. Because the CNS does not enter the blood circulation, the parathyroid will not be dyed black. Therefore, this dye technique can clearly show the parathyroid during the dissection of SLNs and the CCND. It could be a new method for identifying and protecting the parathyroid. However, the false-negative rate of 21.2% (21/99) should be noted. Non-SLN metastases could be caused by the neglected lateral compartment lymph nodes. Additionally, more techniques should be used to promote the efficiency of the pathology department. Then, increasing the accuracy rate of diagnosing micro-metastases in lymph nodes will be helpful.

The predictive factors for central compartment LNMs in PTC patients with occult lymph nodes have not been well defined. However, it is generally accepted that the prognosis depends on sex, tumor multifocality, capsular invasion, and tumor size. This study included several clinicopathological parameters as potential predictors of central compartment LNM. Consequently, male gender, tumor multifocality, and extrathyroidal extension were found to be independent predictors of central compartment SLNM. In addition, male gender was significantly associated with non-SLN metastases. In a series of studies, male gender was associated with higher rates of LNM and was suggested as an important indicator for prophylactic CCND[24–26]. In this study, male gender was significantly associated with SLNMs and non-SLN metastases, which was consistent with previous reports. Patient age is known to be a significant prognostic factor, but in our study, age was not predictive of central compartment LNM; the frequency of subclinical central LNM was slightly greater in patients aged ≤ 45 years. Previous studies have also reported that age was not associated with LNM in PTC[24,27,28]. Generally, LNM is known to increase with tumor size. Yoon et al.[24] reported that tumor size is correlated with central compartment LNM but is not an independent predictor. In this study, the primary tumor size slightly influenced the frequency of central compartment LNM: 42.7% (61/143) for tumors 1 cm or less and 55.1% (38/69) for tumors larger than 1 cm. PTC is often multifocal, and some studies have demonstrated a significant relationship between tumor multifocality and central compartment LNM[24,26]. In this study, tumor multifocality was an independent predictor of SLNM (OR = 2.204, p = 0.035). Extrathyroidal extension is thought to have predictive value for central compartment LNM. In our series, tumoral infiltration of the extrathyroidal tissue was not uncommon (14.2%), which is consistent with previous studies reporting a 9.9% to 26.8% rate [29,30]. It has been demonstrated that extrathyroidal extension is independently predictive of central compartment LNM. Our results indicated that extrathyroidal extension was an independent risk factor for central compartment SLNMs (OR = 2.624, p = 0.033). The association of lymphocytic thyroiditis and aggressive pathologic features of PTC has been debated [31–33]. Previous studies have reported a negative association between the coexistence of lymphocytic thyroiditis and central compartment LNM[34,35]. In this study, we exclusively investigated PTC patients with occult lymph nodes and demonstrated that lymphocytic thyroiditis was not associated with central compartment LNM.

In the present study, the false-negative rate of SLNB should be taken into account. The high false-negative rate would likely lead to missed diagnoses; thus, SLNB using CNS might not be an optimal choice for all patients. In addition, male gender was significantly associated with non-SLN metastases among the PTC patients without SLNM. This result is consistent with previous studies suggesting that male gender is an important indicator for prophylactic CCND[24–26]. Therefore, more attention should be paid to male patients, even those who are SLNM negative, for signs of non-SLN metastases.

However, the present study has potential limitations. We focused on the SLNs in the central compartment, which would have undoubtedly ignored the lateral compartment SLNs. Consequently, some SLNMs in the lateral compartment were missed, and that might be why SLNB yielded the relatively low sensitivity and a high false-negative rate in the present investigation. Moreover, the number of patients included in this study might not have been large enough, especially for the further analyses in the SLN non-metastasis samples. Further investigation of the prognosis with a long follow-up period is necessary. Despite these limitations, we found that SLNB using CNS was conducive not only to guide CCND but also to identify the parathyroid glands during the operation; furthermore, our study was based on variables obtained from patients of the same race and within a local environment. Thus, we believe the present investigation will be useful in the design of further studies.

In conclusion, SLNB using CNS in PTC patients is a technically safe and feasible utility procedure, especially for male patients with co-existing multifocality and extrathyroidal extension. However, before SLNB can be used as a routine procedure and replaces prophylactic CCND, the sensitivity needs to be improved and the false-negative rate of SLNB decreased. We also should pay more attention to PTC patients (especially males) without SLNM for signs of non-SLN metastases.

## Supporting Information

S1 Table Original data for study subjects. (XLS)
